# Prevalence of pharmacogenomic variants in 100 pharmacogenes among Southeast Asian populations under the collaboration of the Southeast Asian Pharmacogenomics Research Network (SEAPharm)

**DOI:** 10.1038/s41439-021-00135-z

**Published:** 2021-02-04

**Authors:** Chakkaphan Runcharoen, Koya Fukunaga, Insee Sensorn, Nareenart Iemwimangsa, Sommon Klumsathian, Hang Tong, Nam Sy Vo, Ly Le, Tin Maung Hlaing, Myo Thant, Shamsul Mohd Zain, Zahurin Mohamed, Yuh-Fen Pung, Francis Capule, Jose Nevado, Catherine Lynn Silao, Zeina N. Al-Mahayri, Bassam R. Ali, Rika Yuliwulandari, Kinasih Prayuni, Hilyatuz Zahroh, Dzul Azri Mohamed Noor, Phonepadith Xangsayarath, Dalouny Xayavong, Sengchanh Kounnavong, Somphou Sayasone, Zoe Kordou, Ioannis Liopetas, Athina Tsikrika, Evangelia-Eirini Tsermpini, Maria Koromina, Christina Mitropoulou, George P. Patrinos, Aumpika Kesornsit, Angkana Charoenyingwattana, Sukanya Wattanapokayakit, Surakameth Mahasirimongkol, Taisei Mushiroda, Wasun Chantratita

**Affiliations:** 1 Center for Medical Genomics, Faculty of Medicine Ramathibodi Hospital, Mahidol University, Bangkok, Thailand; 2 Laboratory for Pharmacogenomics, RIKEN Center for Integrative Medical Sciences, Yokohama, Japan; 3 School of Biotechnology, International University, Vietnam National University, Ho Chi Minh City, Vietnam; 4 Vingroup Big Data Institute, Hanoi, Vietnam; 5 Defence Services Medical Academy, Mingalardon, Myanmar; 6 Defence Services Medical Research Centre, Nay Pyi Taw, Myanmar; 7 Department of Pharmacology, Faculty of Medicine, University of Malaya, Kuala Lumpur, Malaysia; 8 Department of Biomedical Sciences, University of Nottingham (Malaysia Campus), Selangor, Malaysia; 9 Department of Pharmacy, College of Pharmacy, University of the Philippines Manila, Manila, Philippines; 10 Institute of Human Genetics, National Institutes of Health, University of the Philippines Manila, Manila, Philippines; 11 Department of Pediatrics, Philippine General Hospital and College of Medicine, University of the Philippines Manila, Manila, Philippines; 12 Department of Pathology, College of Medicine and Health Sciences, United Arab Emirates University, Al Ain, United Arab Emirates; 13 Department of Pathology and Department of Genetics and Genomics, College of Medicine and Health Sciences, United Arab Emirates University, Al Ain, United Arab Emirates; 14 Department of Pharmacology, Faculty of Medicine, YARSI University, Jakarta, Indonesia; 15 Genetic Research Center, YARSI Research Institute, YARSI University, Jakarta, Indonesia; 16 School of Pharmaceutical Sciences, Universiti Sains Malaysia, Pulau Pinang, Malaysia; 17 National Center for Laboratory and Epidemiology (NCLE), Vientiane, Lao PDR; 18 Lao Tropical and Public Health Institute, Vientiane, Lao PDR; 19 University of Patras, School of Health Sciences, Department of Pharmacy, Laboratory of Pharmacogenomics and Individualised Therapy, Patras, Greece; 20 The Golden Helix Foundation, London, UK; 21 Graduate Program in Molecular Medicine, Faculty of Science, Mahidol University, Bangkok, Thailand; 22 Division of Genomic Medicine and Innovation Support, Department of Medical Sciences, Ministry of Public Health, Nonthaburi, Thailand

**Keywords:** Genetics research, Structural variation

## Abstract

Pharmacogenomics can enhance the outcome of treatment by adopting pharmacogenomic testing to maximize drug efficacy and lower the risk of serious adverse events. Next-generation sequencing (NGS) is a cost-effective technology for genotyping several pharmacogenomic loci at once, thereby increasing publicly available data. A panel of 100 pharmacogenes among Southeast Asian (SEA) populations was resequenced using the NGS platform under the collaboration of the Southeast Asian Pharmacogenomics Research Network (SEAPharm). Here, we present the frequencies of pharmacogenomic variants and the comparison of these pharmacogenomic variants among different SEA populations and other populations used as controls. We investigated the different types of pharmacogenomic variants, especially those that may have a functional impact. Our results provide substantial genetic variations at 100 pharmacogenomic loci among SEA populations that may contribute to interpopulation variability in drug response phenotypes. Correspondingly, this study provides basic information for further pharmacogenomic investigations in SEA populations.

Pharmacogenomics is the study of how an individual’s genomic profile influences their response to drug treatments. This has emerged as a potential tool to optimize medications and reduce adverse drug events^1^. Genotyping data from next-generation sequencing (NGS) technologies are increasing in international public databases, thereby enabling new advances in pharmacogenomic research. Implementation guidelines of the data are now available from organizations such as the Clinical Pharmacogenetics Implementation Consortium (CPIC)^2^.

The Southeast Asian Pharmacogenomics Research Network (SEAPharm) was founded in 2012. SEAPharm aims to be the regional pharmacogenomics (PGx) network to strengthen the knowledge of PGx research and its implementation approaches in SEA countries^3^. In 2018, the annual SEAPharm meeting approved an expanded research collaboration under the project entitled “Re-sequencing Project of 1,000 Southeast Asian Individuals Using the 100 Pharmacogene - Next Generation Sequencing Panel” using the NGS platform. Nine countries participated in this project: seven countries from Southeast Asia (Indonesia, Laos, Malaysia, Myanmar, Philippines, Thailand, and Vietnam) and one each from Europe (Greece) and Western Asia (United Arab Emirates; UAE).

The 100 PKSeq panel is composed of 37 drug transporter genes, 30 cytochrome P450 (CYP) enzyme-encoded genes, 10 uridine diphosphate glucuronosyltransferase (UGT) genes, 5 flavin-containing monooxygenase (FMO) genes, 4 glutathione S-transferase (GST) genes, 4 sulfotransferase (SULT) genes, and others^4^. Initially, the 100 pharmacogene resequencing processes were performed by RIKEN, Japan. Genomic DNA samples were collected from nine countries: Indonesia (*N* = 562), Laos (*N* = 100), Malaysia (*N* = 105), Myanmar (*N* = 100), the Philippines (*N* = 100), Thailand (*N* = 100), Vietnam (*N* = 100), the UAE (*N* = 100), and Greece (*N* = 304). The latter two populations were used as a control for the former seven populations. Targeted resequencing processes were performed as described previously^4^. After sequencing, the raw data (.fastq files) were further analyzed by the Center for Medical Genomics, Thailand, for primary sequence analysis. Sequencing reads were aligned to the human reference genome (GRCh37/hg19) by using the Burrows-Wheeler Aligner (0.7.17). Variants, including single-nucleotide polymorphisms (SNPs) and short insertions and deletions (indels), were called using the Genome Analysis Toolkit (GATK, v3.5)^5^. Variant quality score recalibration (VQSR) was also applied as call set refinement to reduce the number of false-positive calls. BCFtools was used to manipulate the variant calling format (.vcf) files and to calculate the pairwise weighted *F*_st_ value. Linkage disequilibrium (LD) plots were created by LDBlockShow (Supplementary Figs. 1–13)^6^. Downstream variant annotation and statistical analysis, including plotting, were performed using VarSeq (Golden Helix, Inc., Bozeman, MT, USA, www.goldenhelix.com.) and R software (R Foundation for Statistical Computing, Vienna, Austria, www.R-project.org).

In this report, the frequencies of pharmacogenomic variants in SEA populations based on the 100 PKSeq panel are reported (Supplementary Tables 1–7). Based on the variant calling processes, 3527 variants were called and passed for VQSR processes. In total, 306 variants (excluding multiallelic variants) were jointly observed in the SEA populations (Supplementary Table 8). To quantify the differences in frequencies between this dataset and the public genome dataset, the frequencies of the 306 variants identified in this dataset and in the East Asian (EAS) control datasets from the Genome Aggregation Database (gnomAD v.2.1.1) were compared using scatter plots and correlation coefficient analysis (Fig. 1a). The results revealed concordance between the allelic frequencies in this dataset (in all the SEA populations) and in the EAS dataset from gnomAD. Considering the correlation coefficient R, there was a trend of high correlations in the frequencies of variants in the Thailand, Vietnam, and EAS datasets. To investigate the proportion of the total pharmacogenomic variants contained in SEA populations, pairwise *F*_st_ statistics of the seven SEA countries, UAE, and Greece were performed. The results indicated that the SEA populations had modest genetic similarity (pairwise *F*_st_ value <0.05; Fig. 1b). The greatest genetic similarity was observed in the mainland SEA populations based on their pharmacogenomic background. The Malaysian population seemed to share more similarities with other SEA populations, with the Philippines showing the least similarities among the SEA neighbors. To our knowledge, this is the first report to compare the total pharmacogenomic variants between SEA populations based on the 100 PKSeq panel. A previous study demonstrated the comparison of pairwise *F*_st_ values between Singapore Genome Variation Project (SGVP) populations (Chinese (CHS), Indian (INS), and Malay (MAS)), South Asians (SAS), and Europeans by using the variants of ADME^7^. The results showed that the CHS and MAS populations were profoundly different from the SAS and INS populations, which exhibited substantial similarity^7^.

**Fig. 1:**
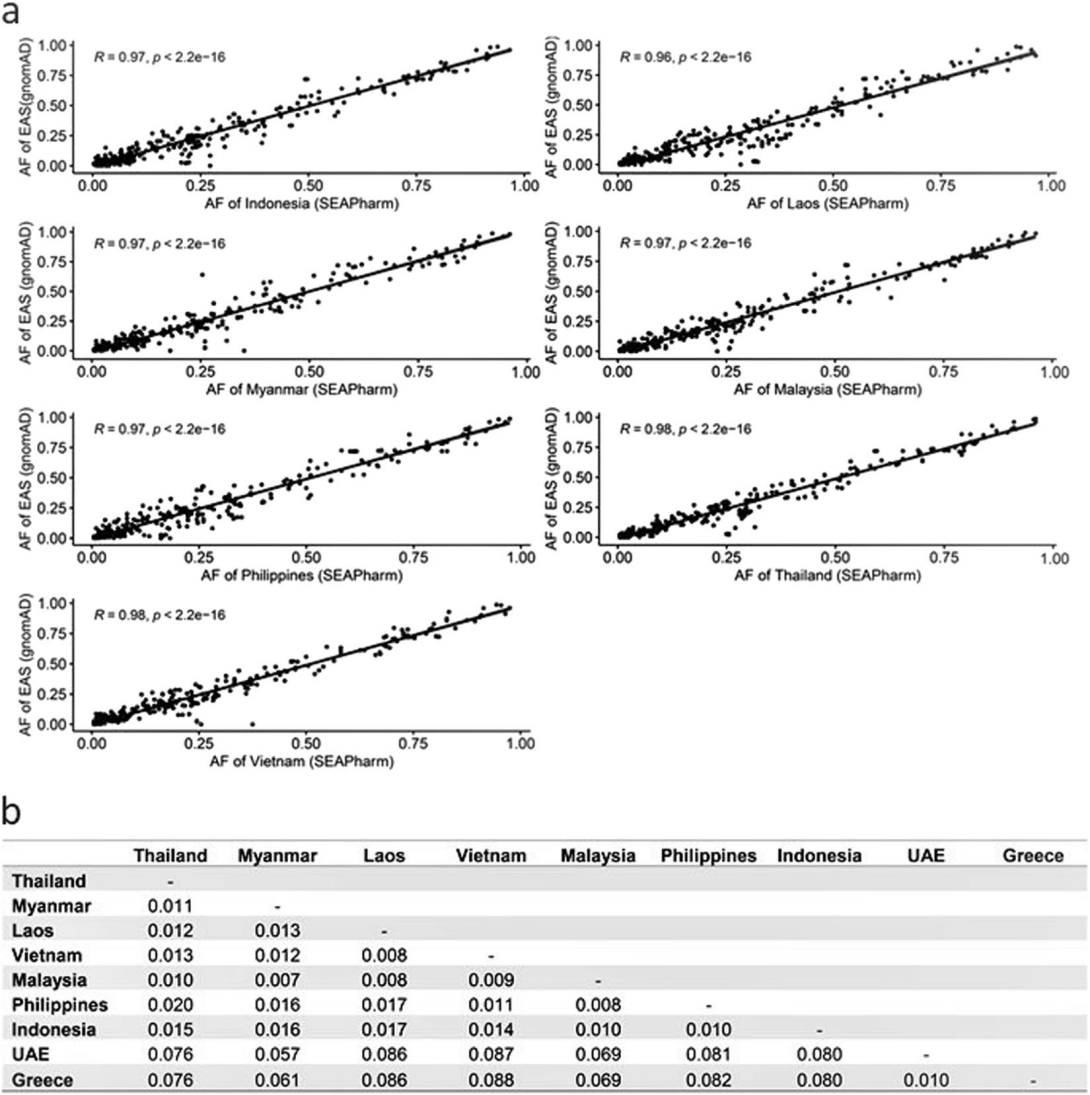
Scatter plot and correlation coefficient of this dataset and EAS datasets from gnomAD and pairwise weighted *F*_st_ statistics between SEA populations. **a** Frequencies of the 306 pharmacogenomic variants between this dataset and East Asian (EAS) datasets from gnomAD. AF Allele frequency. **b** Pairwise weighted *F*_st_ statistics between SEA, UAE, and Greek populations.

The allele frequencies of the genes responsible for drug metabolism enzymes and transporters in the SEA populations were then explored^8,9^. The frequencies of the variants of *CYP2C9*, *CYP2C19*, *CYP2D6*, *CYP3A4*, *ABCB1*, *ABCG2*, *SLC22A2*, *SLC22A6*, *SLC22A8*, *SLCO1B3*, and *SLCO1B3*, which were jointly observed in the SEA populations, are shown in Fig. 2a. Two variants of *CYP2D6* differed in the observed minor allele frequencies (MAFs), with an allele frequency <0.5, between the SEA populations. The MAF of *CYP2D6* rs1065852 (A) was 0.410 in and 0.495 in the Burmese and Malaysian populations. The frequency of the A allele of *CYP2D6* rs1081003 was 0.395, 0.490, and 0.492 in the Burmese, Malaysian, and Indonesian populations; however, allele A was found to be a major allele in other SEA populations. As previously described, the frequencies of pharmacogenomic variants among the sample population from Myanmar residing in the USA demonstrated that the MAF of rs1065852 was higher in this population (A) (MAF = 0.36) than in the American (AMR, MAF = 0.15) and South Asian (SAS, MAF = 0.16) populations and slightly lower than that in the EAS population^10^. rs1065852 (A) is recognized as a key mutation in CYP2D6*10. In Malaysia, the allelic frequencies of CYP2D6*10 differed between the Chinese-Malaysians, Malay-Malaysians, and Indian-Malaysians^11^. Only the MAF of the CYP2D6*10 allele was noted in the Indian-Malaysians (MAF = 0.214). However, the frequencies of rs1065852 (A) are not represented for *CYP2D6* copy number variation (CNV)-variable haplotypes such as CYP2D6*36. The frequency of rs1081003 (A) was 0.412 among the Chinese population. The frequencies also varied among subpopulations (i.e., Shanghai (MAF = 0.484), Xi’an (MAF = 0.407), Shenyang (MAF = 0.467), and Shantou (MAF = 0.288))^12^. In addition, this variant has been reported as a major allele in Taiwanese^13^. The LD plot of *CYP2D6* in the population revealed that five SNPs (rs1135840, rs16947, rs1058164, rs1081003, and rs1065852) in the Philippinean, Thailand, and Vietnamese populations and three SNPs (rs1058164, rs1081003, and rs1065852) in the Laos population were in very high LD (Supplementary Fig. 13). These SNPs are key mutations in CYP2D6*10A and CYP2D6*54 and cause a decrease in enzyme activity. As previously described, CYP2D6*10 is responsible for the intermediate metabolizer status in SEA populations. However, the prevalence of these alleles is low in Malay-Singaporeans, Chinese-Singaporeans, Indian-Malaysians, and Indian-Singaporeans^14^. Additionally, two variants of the drug transporter genes, namely, rs1128503 (G) and rs2291075 (T) of *ABCB1* and *SLCO1B1*, respectively, differed in the observed allele frequencies between populations (Fig. 2b). Interestingly, some *ABCB1* variants seem to be more frequent in Filipinos, such as rs1045642 (G), with an allele frequency of 0.71. However, *ABCB1* rs1128503 (G), a minor allele in other SEA populations, was found to be a major allele among Filipinos. Previously, rs1128503 (G) was documented as a major allele among Chinese and Singaporean populations; however, rs1128503 (G) was found to be a minor allele in the Indonesian population^15,16^. rs1128503, rs2032582, and rs1045642 are the most common SNPs in the coding region of *ABCB1*; moreover, these SNPs are in strong LD^17^. The homozygous variants for one of three *ABCB1* variants, rs1128503 (T), rs2032582 (T, A), and rs1045642 (T), are associated with significantly high short-term remission rates after tacrolimus treatment in steroid-refractory ulcerative colitis (UC) patients^18^. The MAFs of rs2291075 (T) are 0.400 and 0.470 in the Burmese and Vietnamese populations. In Singapore, the frequencies of rs2291075 (T) vary between subpopulations (CHS, INS, and MAS); nonetheless, the MAF was observed only in INS (MAF = 0.031)^19^. Moreover, rs2291075 (T) was previously described as a MAF in Korean (MAF = 0.436) and Japanese (MAF = 0.367) populations. In contrast, this variant was found to be a major allele among the Chinese population^20^. rs2291075, which encodes the transporter OATP1B1, is in strong LD with rs2306283 and rs4149056. Inheritance of variability in the transporter OATP1B1 may influence the effectiveness of acute myeloid leukemia (AML) therapy because this transporter is responsible for the systemic pharmacokinetics of several drugs used in AML treatment^21^.

**Fig. 2:**
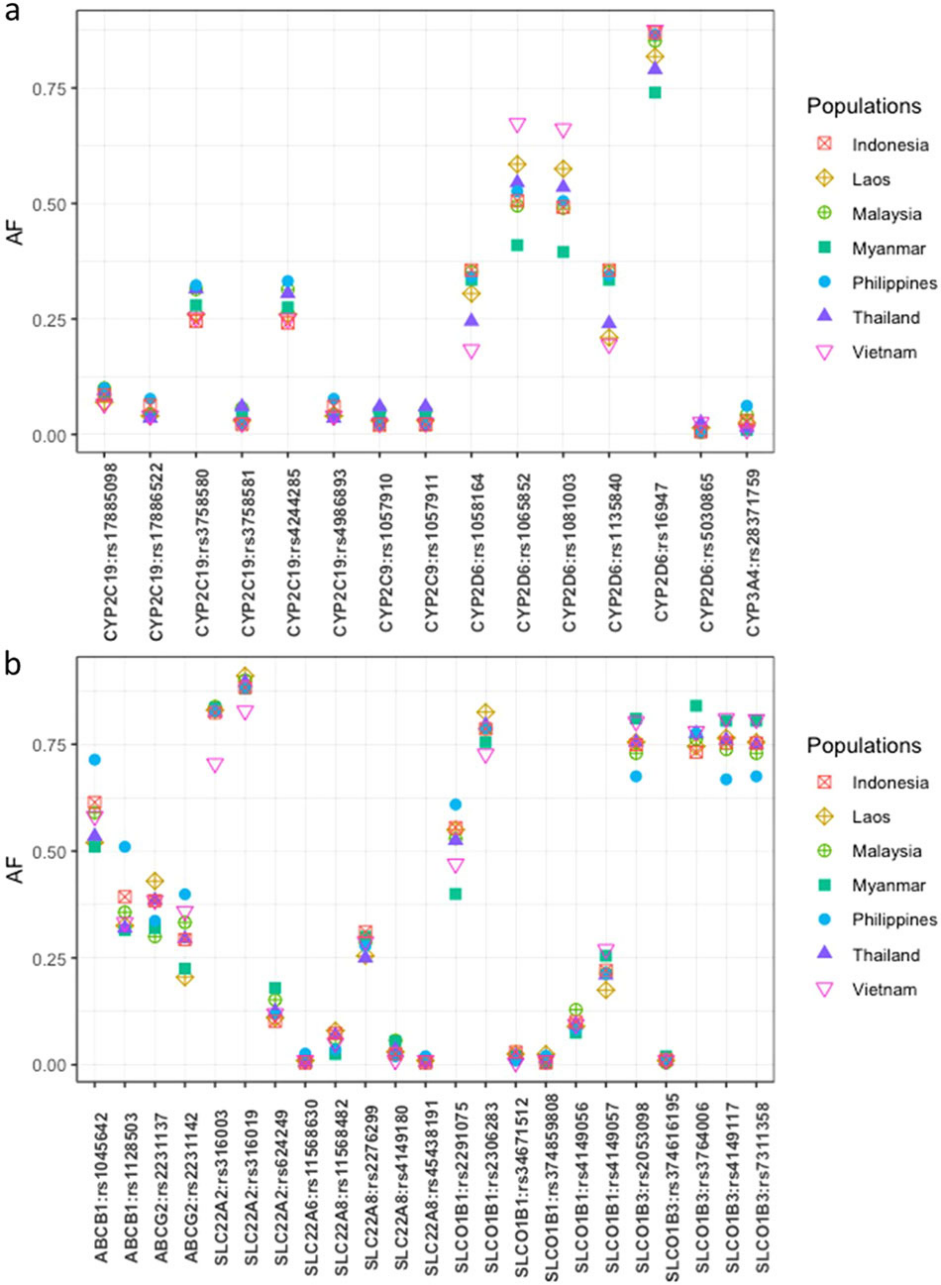
Comparison of the frequencies of major genes responsible for drug metabolism enzymes and transporters in the SEA populations. a) Frequencies of pharmacogenomic variants of *CYP2C9*, *CYP2C19*, *CYP2D6*, and *CYP3A4*. b) Frequencies of pharmacogenomic variants of *ABCB1*, *ABCG2*, *SLC22A2*, *SLC22A6*, *SLC22A8*, *SLCO1B3* and *SLCO1B3*. AF, Allele frequency.

Although the SEA populations seem to have genetic similarities, differences in allele frequencies between the populations were observed. A previous large-scale study of pharmacogenomic biomarkers in 18 European populations demonstrated allele frequency differences in the interpopulations^22^. Additionally, the genotyping of CYP genes across Native American and Ibero-American populations revealed differences within Native Americans^23^. These findings support that ethnicity affects differences in drug response and/or toxicity. To support the need for personalized precision medicine, the interethnic differences of SEA populations should be taken into consideration to reliably predict drug safety and efficacy at the population level.

We further investigated the functional impact of these pharmacogenomic variants, including deleterious missense mutations and loss-of-function mutations, among the SEA populations. Deleterious missense mutations were determined by multiple algorithms from a database for the functional predictions of nonsynonymous SNPs (dbNSFP v3.0) via VarSeq (Golden Helix, Inc., Bozeman, MT, USA, www.goldenhelix.com). Considering the functional impacts of the variants (i.e., major allele frequencies and MAFs), there was a trend of higher proportions of deleterious missense and loss-of-function mutations in the MAF variants (Supplementary Fig. 14). In particular, rare variants (MAF < 0.01) accounted for the highest proportions of deleterious missense and loss-of-function mutations. Whole-genome sequencing of Malaysians revealed 693 variants of 8550 predicted deleterious variants in 437 pharmacogenomic genes involved in drug metabolism. Almost seventy percent (70%) of the variants were rare alleles^24^. The investigation of variants in 12 CYP genes revealed that the majority of variants are remarkably rare in both African-American and European-American ancestries^25^. Additionally, a large proportion of rare alleles with the potential to impact drug metabolism has been documented in Slovenian and Latino populations^26,27^. This is also supported by the investigation of individual variants by sequencing drug target genes, which demonstrated that rare variants are abundant in humans, and many have potentially relevant effects on drug metabolism^25,28,29^. Rare variants of pharmacogenes are significantly associated with variations that contribute to a significant portion of the unexplained interindividual differences in drug metabolism phenotypes, thereby causing functional alterations^28,30^.

In conclusion, this report presents the data on the frequencies of 100 pharmacogenes from the 100 PKSeq resequencing panel. We reported the frequencies of the pharmacogenomic variants and compared the pharmacogenomic variants among different SEA populations. Additionally, we examined the functional impact of the pharmacogenomic variants that potentially caused functional alterations. These data provide a useful resource for future pharmacogenomic research in SEA populations.

## Supplementary information

Supplementary Figure 1–14

Supplementary Table 1

Supplementary Table 2

Supplementary Table 5

Supplementary Table 6

Supplementary Table 7

Supplementary Table 3

Supplementary Table 4

Supplementary Table 8

## Supplementary information

**Supplementary information** The online version contains supplementary material available at 10.1038/s41439-021-00135-z.
