# Supplementary material for: Prevalence of pharmacogenomic variants in 100 pharmacogenes among Southeast Asian populations under the collaboration of the Southeast Asian Pharmacogenomics Research Network (SEAPharm)
Source: Hum Genome Var. 2021 Feb 4;8:7. doi: 10.1038/s41439-021-00135-z (PMC7862625; doi:10.1038/s41439-021-00135-z)
Supplement: Supplementary file 1 — Supplementary Figure 1–14 [file 41439_2021_135_MOESM1_ESM.docx]

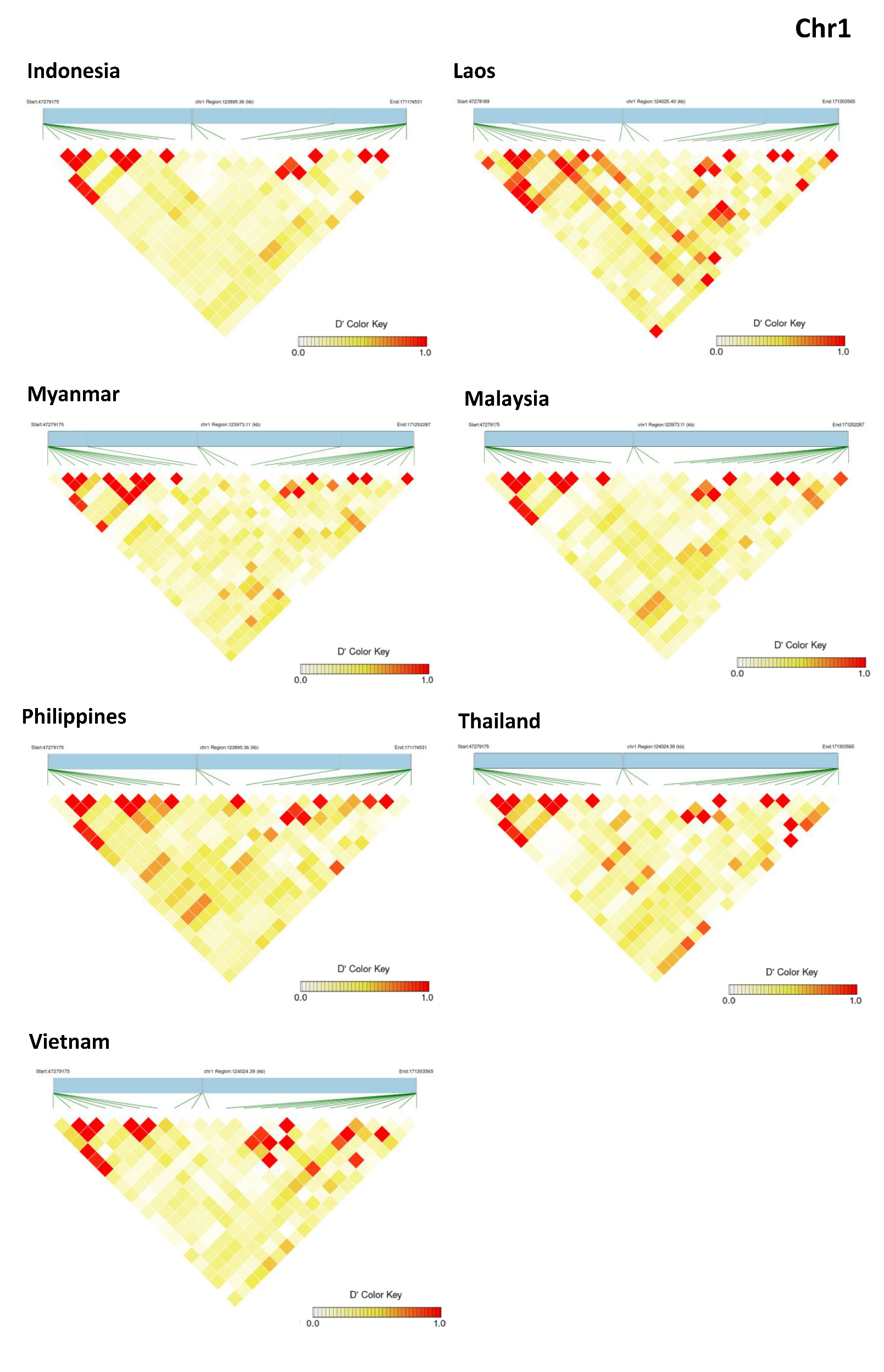


**Supplementary Figure 1**. Linkage disequilibrium plot of the variants within Chromosome 1 in SEA populations. Linkage disequilibrium for SNPs relative within Chromosome 1 (Chr1:47279175-171303565) of seven countries denotes D’ value. There are eight genes of the 100PKSeq panel located in this region including *CYP4B1*, *CYP4A11*, *CYP4Z1*, *DPYD*, *FMO3*, *FMO2*, *FMO1* and *FMO4*.


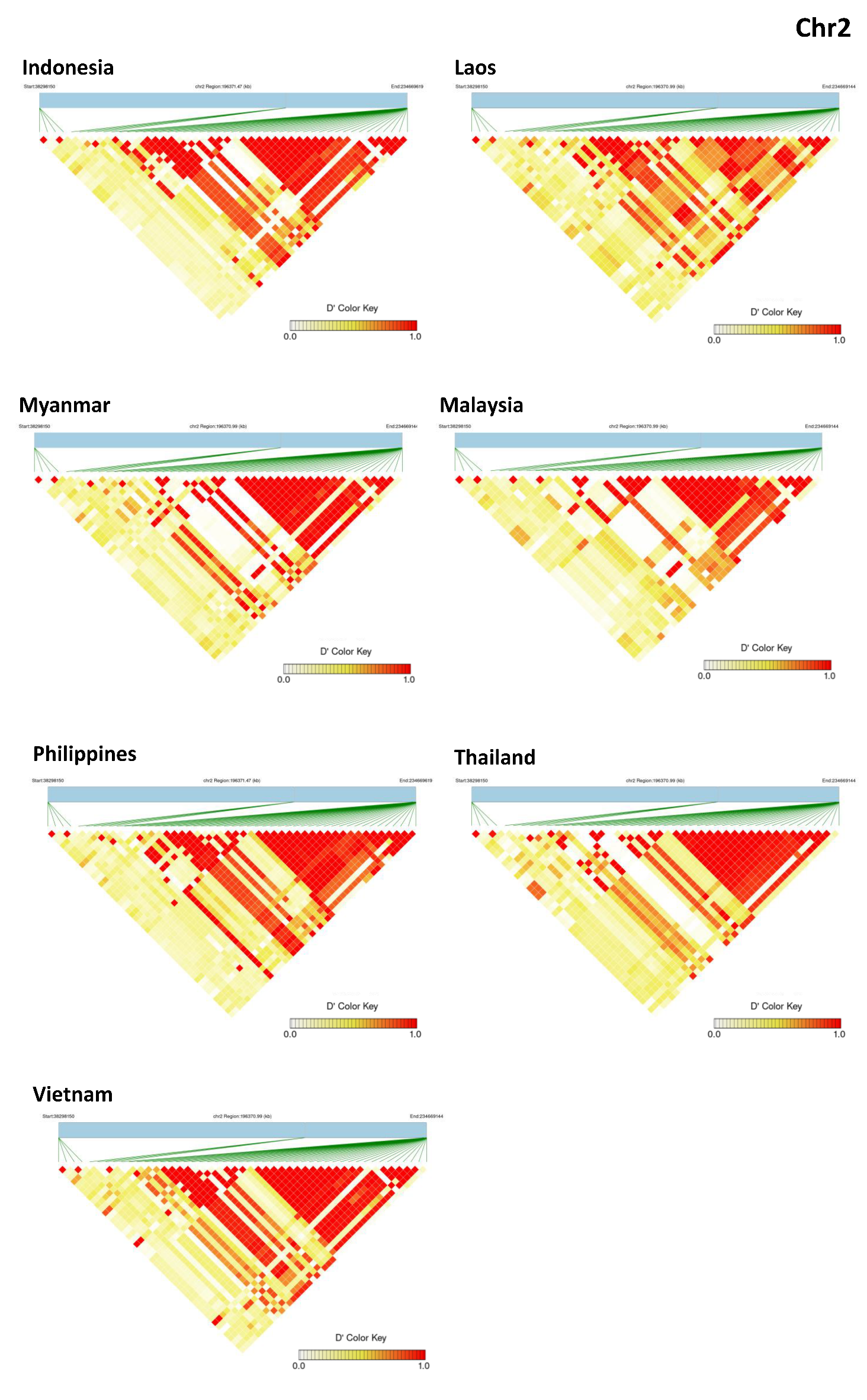


**Supplementary Figure 2**. Linkage disequilibrium plot of the variants within Chromosome 2 in SEA populations. Linkage disequilibrium for SNPs relative within Chromosome 2 (Chr2:38298150-234669144) of seven countries denotes D’ value. There are eleven genes of the 100PKSeq panel located in this region including *CYP1B1*, *ABCB11*, *UGT1A1*, *UGT1A3*, *UGT1A4*, *UGT1A5*, *UGT1A6*, *UGT1A7*, *UGT1A8*, *UGT1A9* and *UGT1A10*.


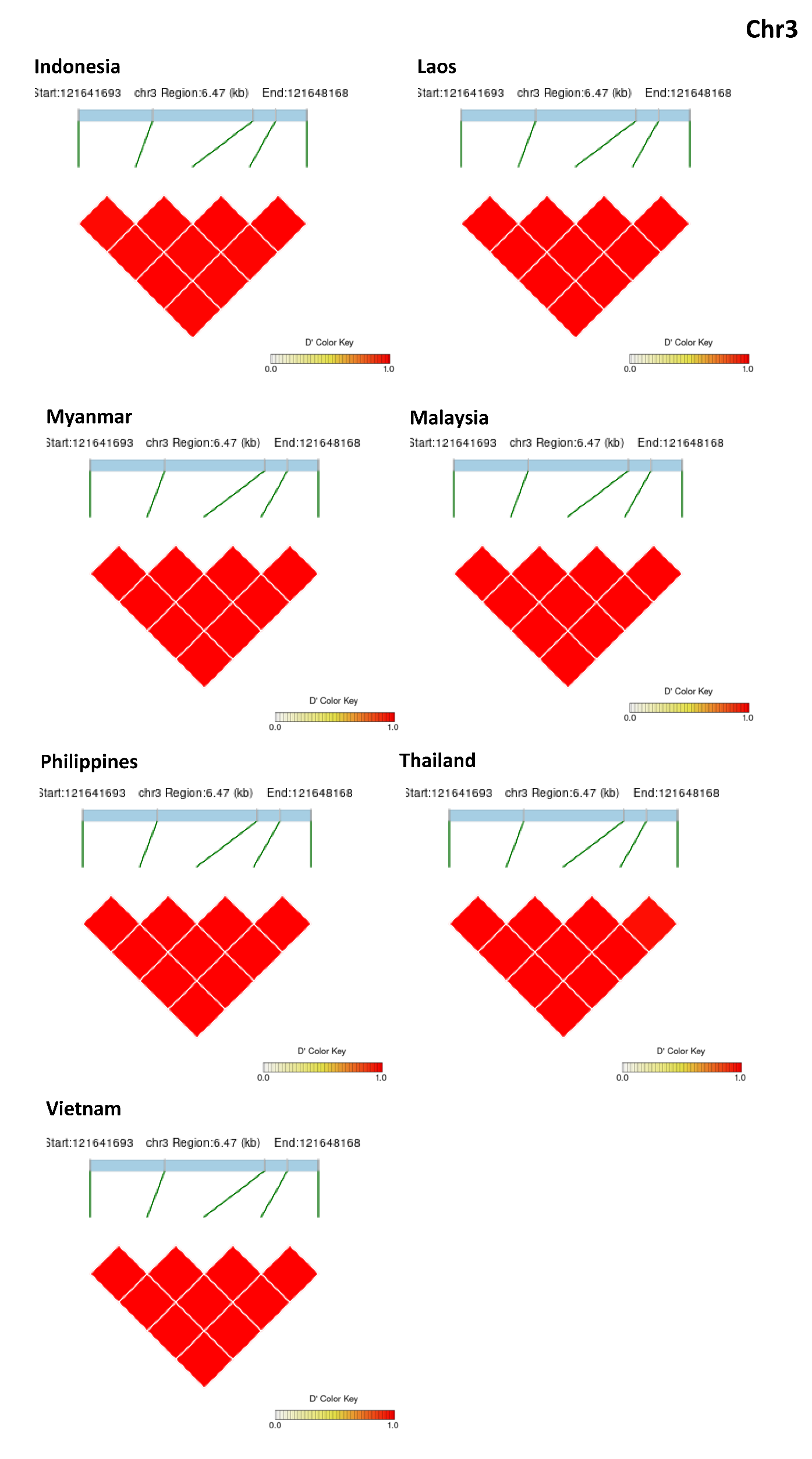


**Supplementary Figure 3**. Linkage disequilibrium plot of the variants within Chromosome 3 in SEA populations. Linkage disequilibrium for SNPs relative within Chromosome 3 (Chr3:121641693-121648168) of seven countries denotes D’ value. There are five SNPs including rs2293616, rs2257212, rs1143670, rs1143671 and rs1143672 of *SLC15A2* located in this region.


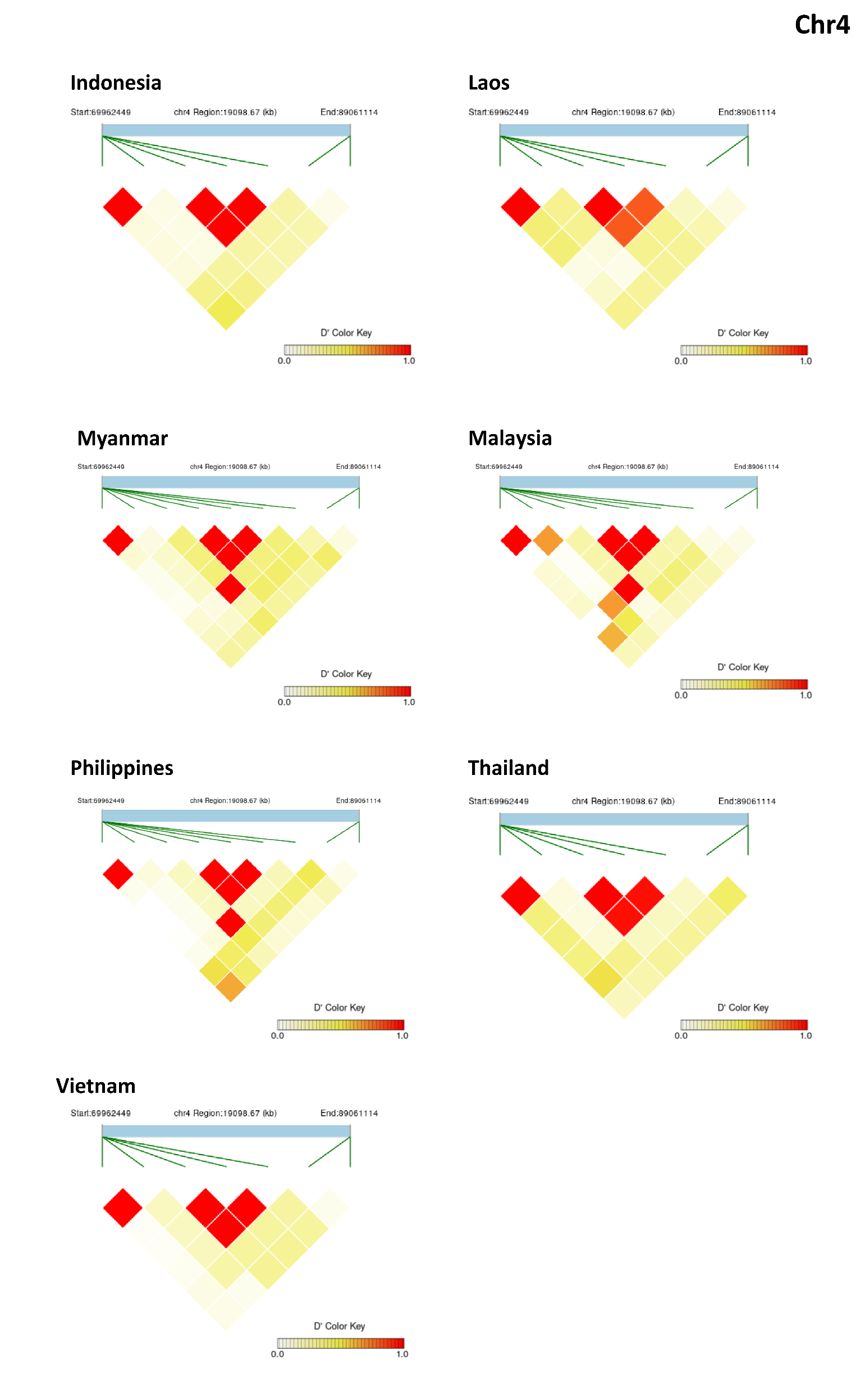


**Supplementary Figure 4**. Linkage disequilibrium plot of the variants within Chromosome 4 in SEA populations. Linkage disequilibrium for SNPs relative within Chromosome 4 (Chr4:69962449-89061114) of seven countries denotes D’ value. There are two genes of the 100PKSeq panel located in this region including *UGT2B7* and *ABCG2*.


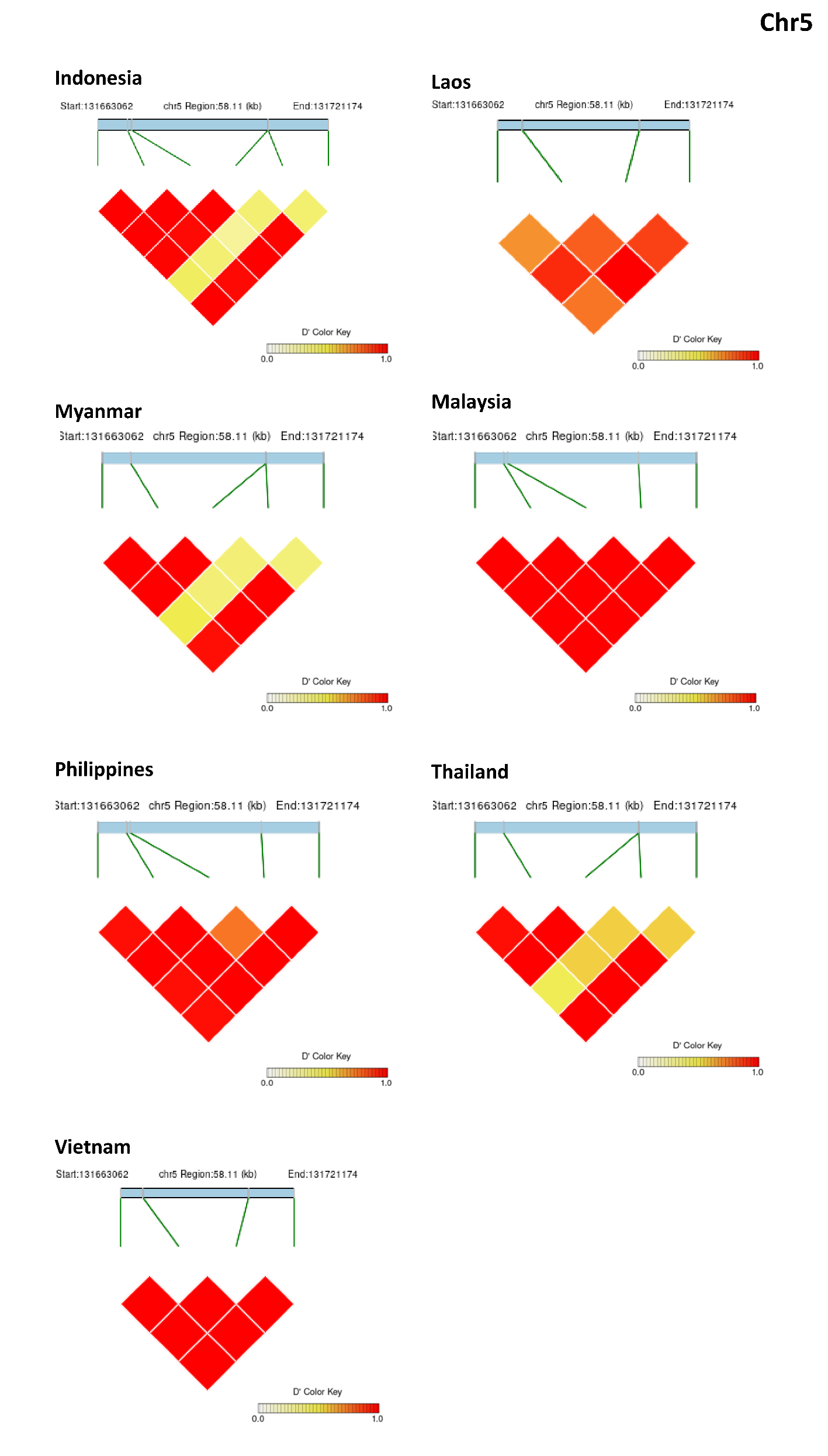


**Supplementary Figure 5**. Linkage disequilibrium plot of the variants within Chromosome 5 in SEA populations. Linkage disequilibrium for SNPs relative within Chromosome 5 (Chr5:131663062-131721174) of seven countries denotes D’ value. There are two genes of the 100PKSeq panel located in this region including *SLC22A4* and *SLC22A5*.


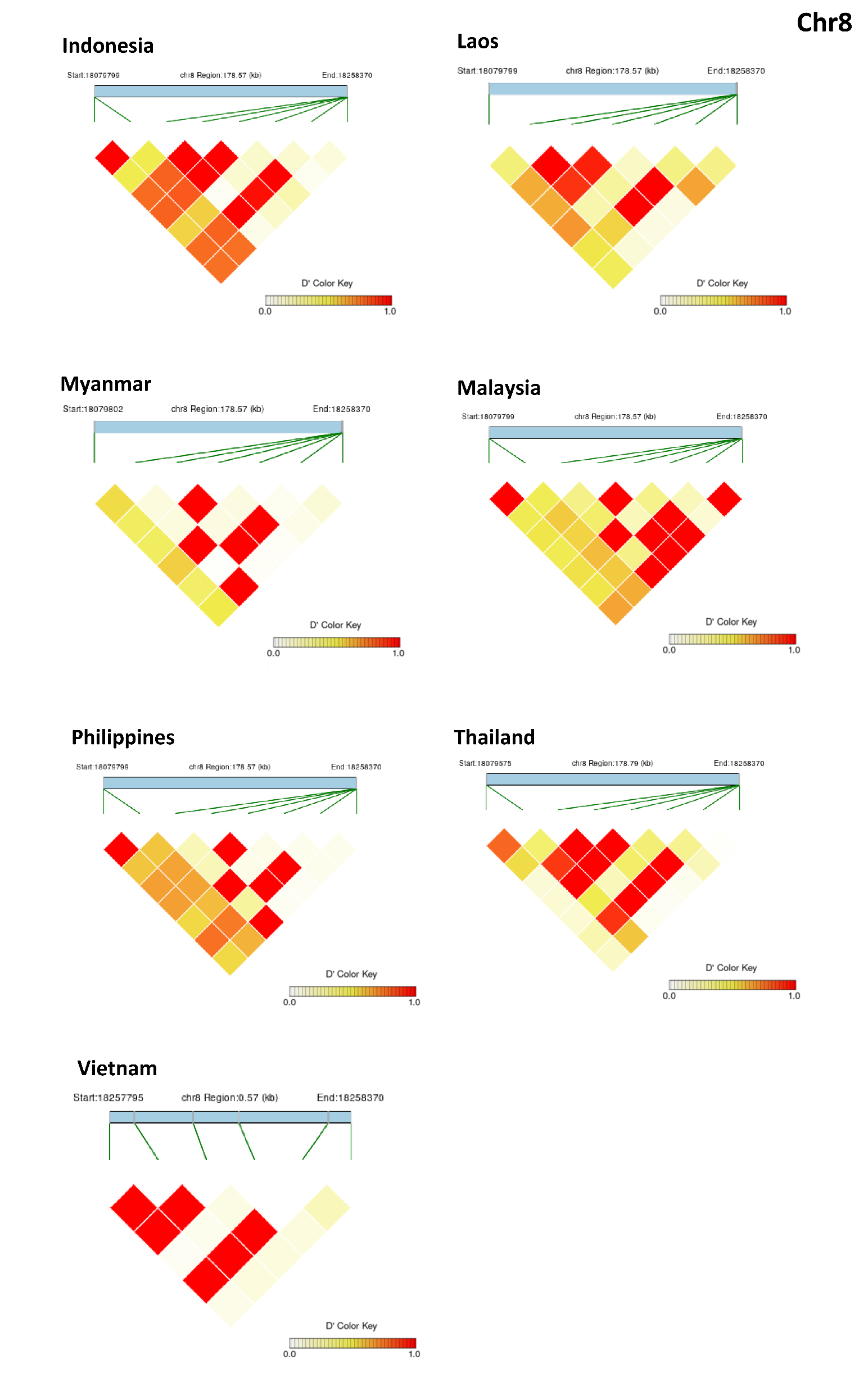


**Supplementary Figure 6**. Linkage disequilibrium plot of the variants within Chromosome 8 in SEA populations. Linkage disequilibrium for SNPs relative within Chromosome 8 (Chr8:18079575-18258370) of seven countries denotes D’ value. There are two genes of the 100PKSeq panel located in this region including *NAT1* and *NAT2*.


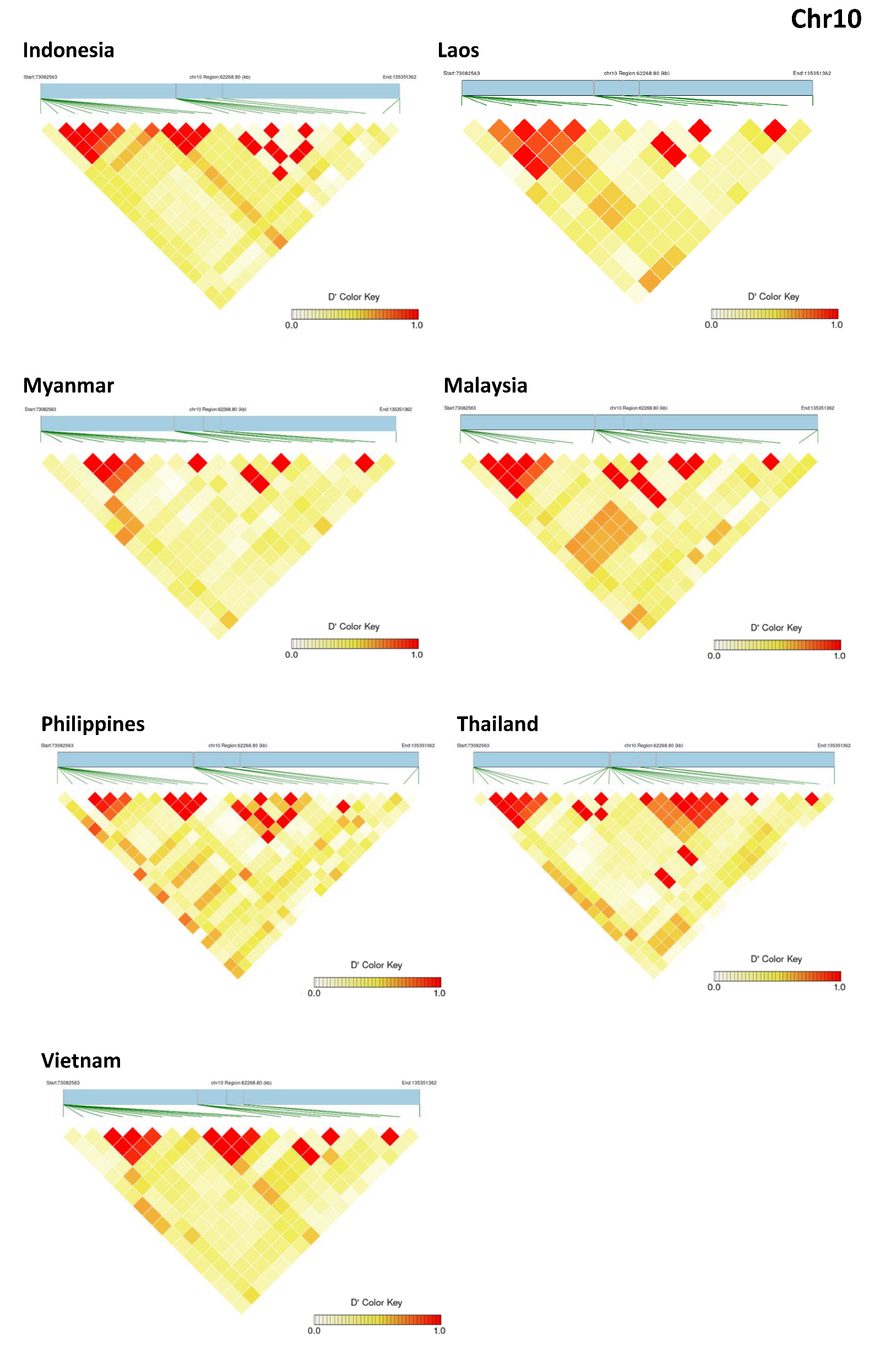


**Supplementary Figure 7**. Linkage disequilibrium plot of the variants within Chromosome 10 in SEA populations. Linkage disequilibrium for SNPs relative within Chromosome 10 (Chr10:73082563-135351362) of seven countries denotes D’ value. There are seven genes of the 100PKSeq panel located in this region including *SLC29A3*, *CYP2C18*, *CYP2C19*, *CYP2C9*, *ABCC2*, *CYP17A1* and *CYP2E1*.


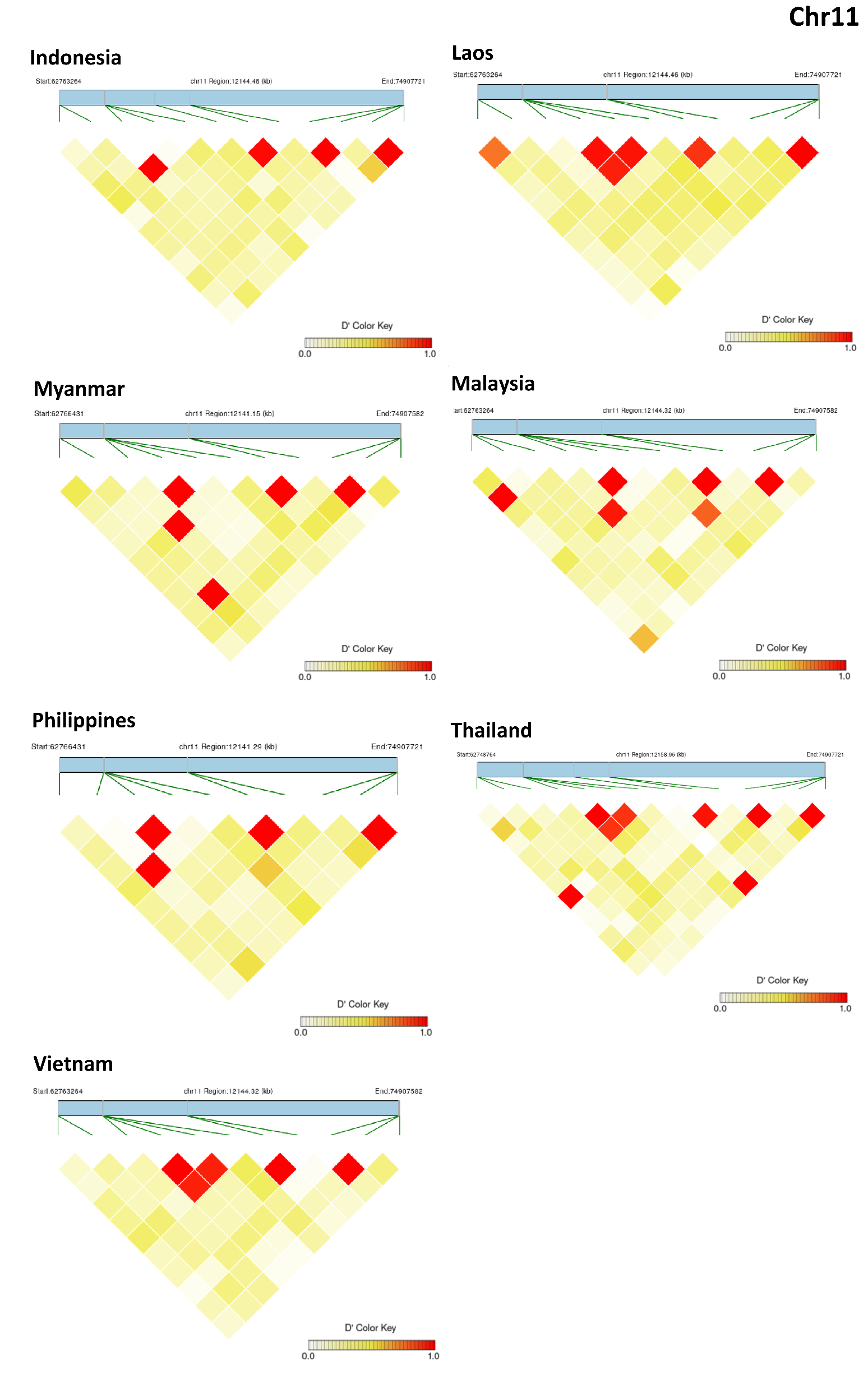


**Supplementary Figure 8**. Linkage disequilibrium plot of the variants within Chromosome 11 in SEA populations. Linkage disequilibrium for SNPs relative within Chromosome 11 (Chr11:62748764-74907721) of seven countries denotes D’ value. There are six genes of the 100PKSeq panel located in this region including *SLC22A6*, *SLC22A8, SLC22A12*, *SLC29A2*, *GSTP1* and *SLCO2B1*.


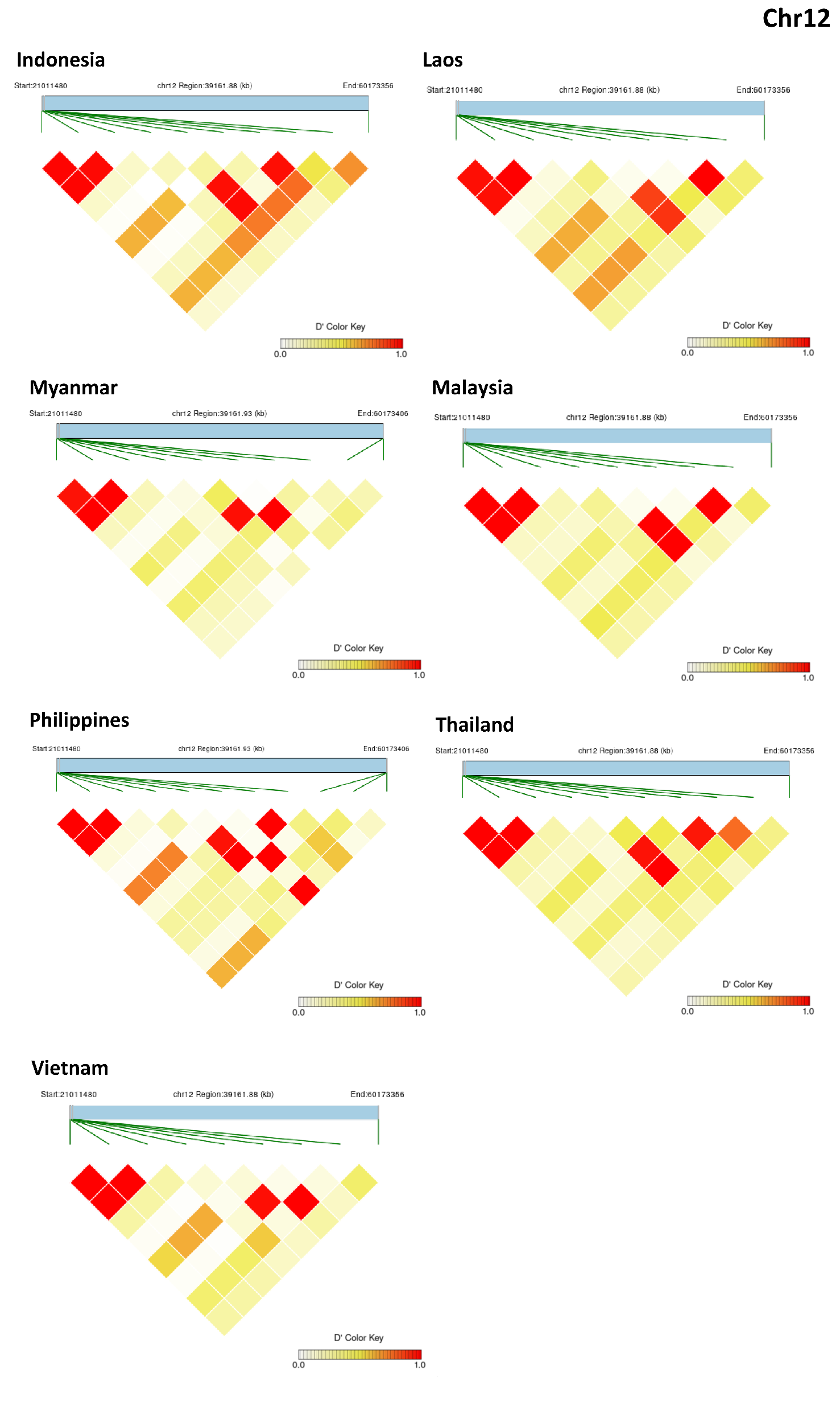


**Supplementary Figure 9**. Linkage disequilibrium plot of the variants within Chromosome 12 in SEA populations. Linkage disequilibrium for SNPs relative within Chromosome 12 (Chr12:21011480-60173356) of seven countries denotes D’ value. There are three genes of the 100PKSeq panel located in this region including *SLCO1B3*, *SLCO1B1* and *SLC16A7.*


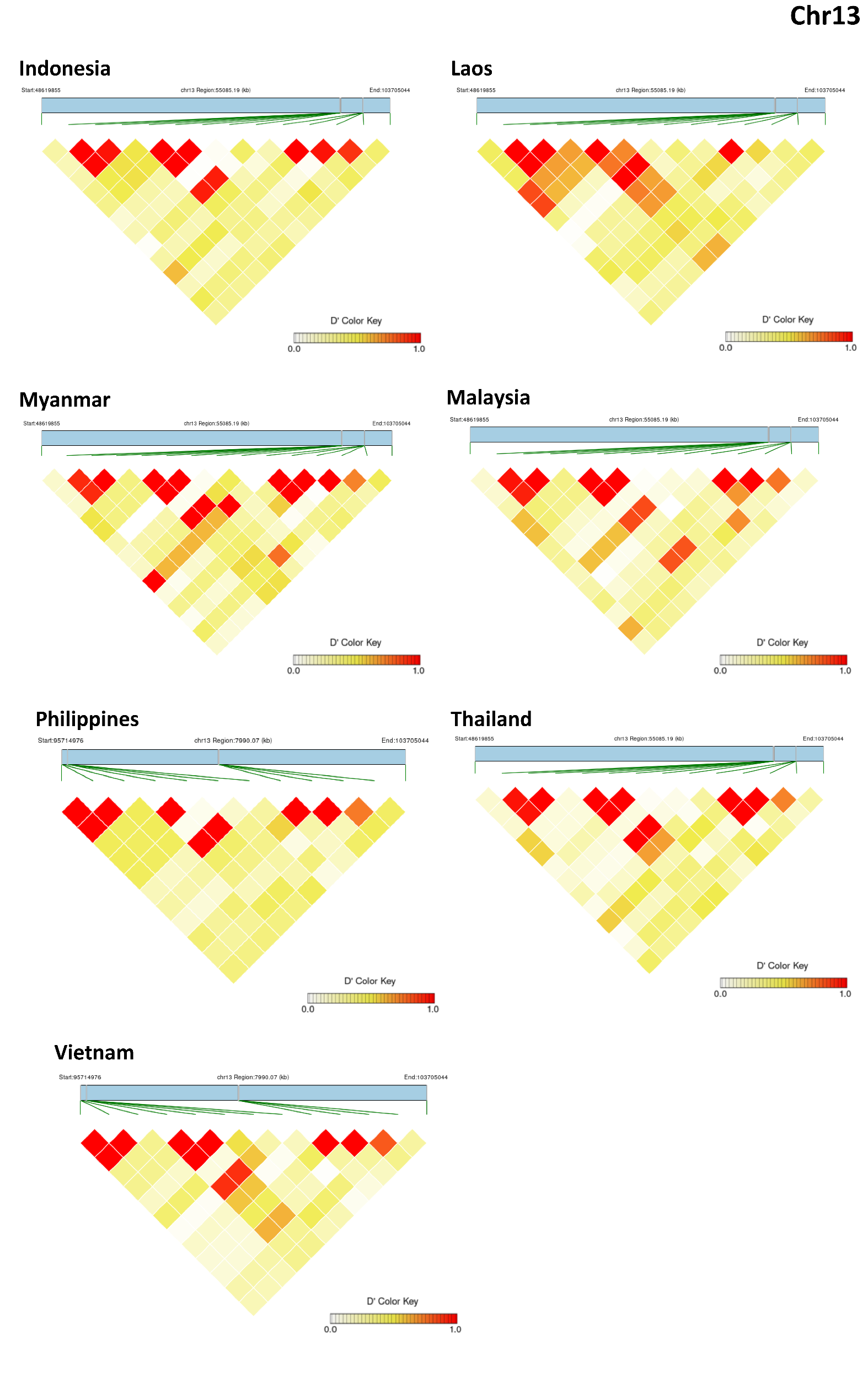


**Supplementary Figure 10**. Linkage disequilibrium plot of the variants within Chromosome 13 in SEA populations. Linkage disequilibrium for SNPs relative within Chromosome 13 (Chr13:48619855-103705044) of seven countries denotes D’ value. There are four genes of the 100PKSeq panel located in this region including *NUDT15*, *ABCC4*, *SLC15A1* and *SLC10A2*.


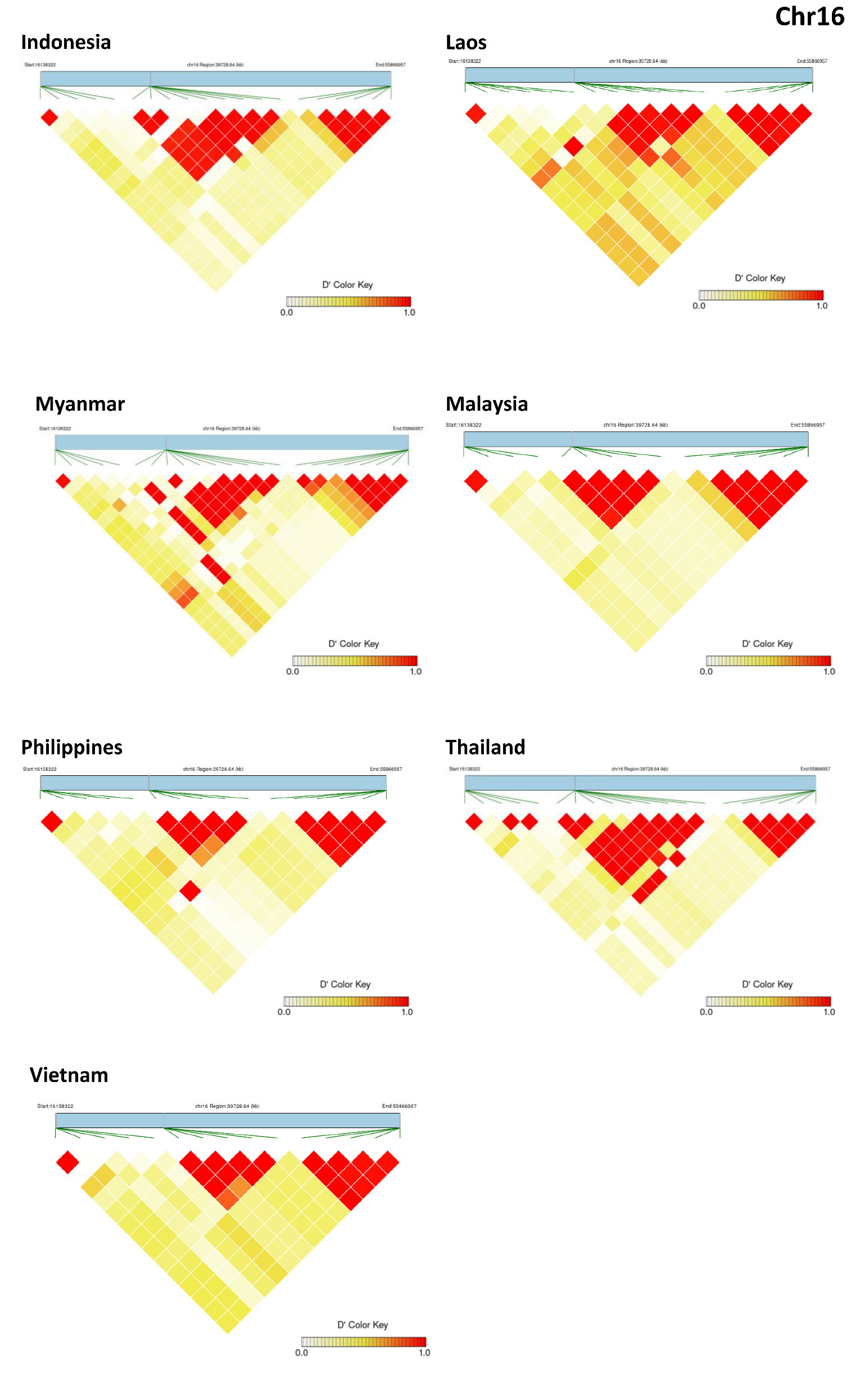


**Supplementary Figure 11**. Linkage disequilibrium plot of the variants within Chromosome 16 in SEA populations. Linkage disequilibrium for SNPs relative within Chromosome 16 (Chr16:16138322-55866957) of seven countries denotes D’ value. There are four genes of the 100PKSeq panel located in this region including *ABCC1*, *SULT1A2, SULT1A1* and *CES1*.


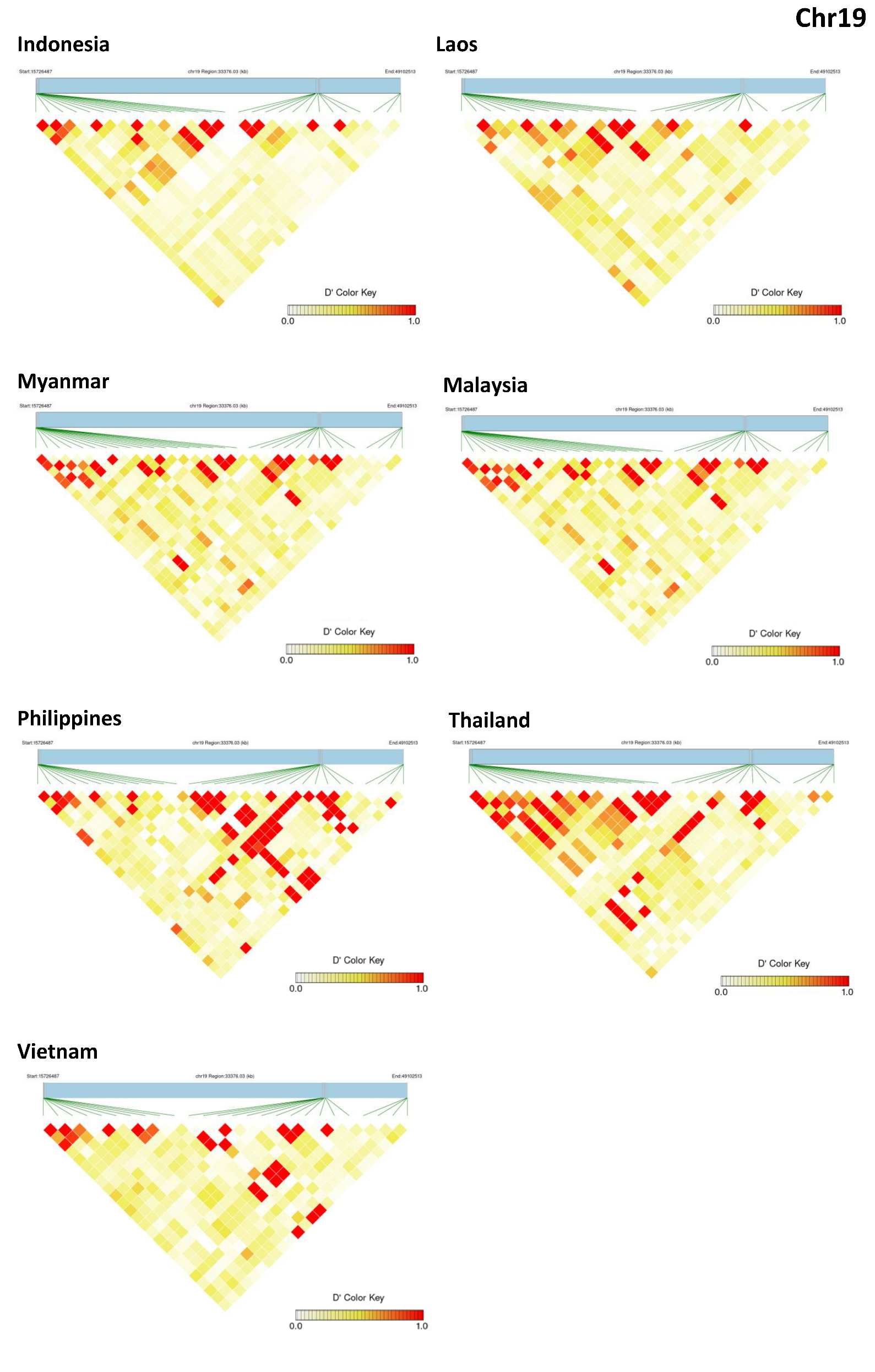


**Supplementary Figure 12**. Linkage disequilibrium plot of the variants within Chromosome 19 in SEA populations. Linkage disequilibrium for SNPs relative within Chromosome 19 (Chr19:15726487-49102513) of seven countries denotes D’ value. There are eight genes of the 100PKSeq panel located in this region including *CYP4F8*, *CYP4F3, CYP4F12, CYP2A6, CYP2B6, CYP2A13, CYP2S1* and *SULT2B1*.


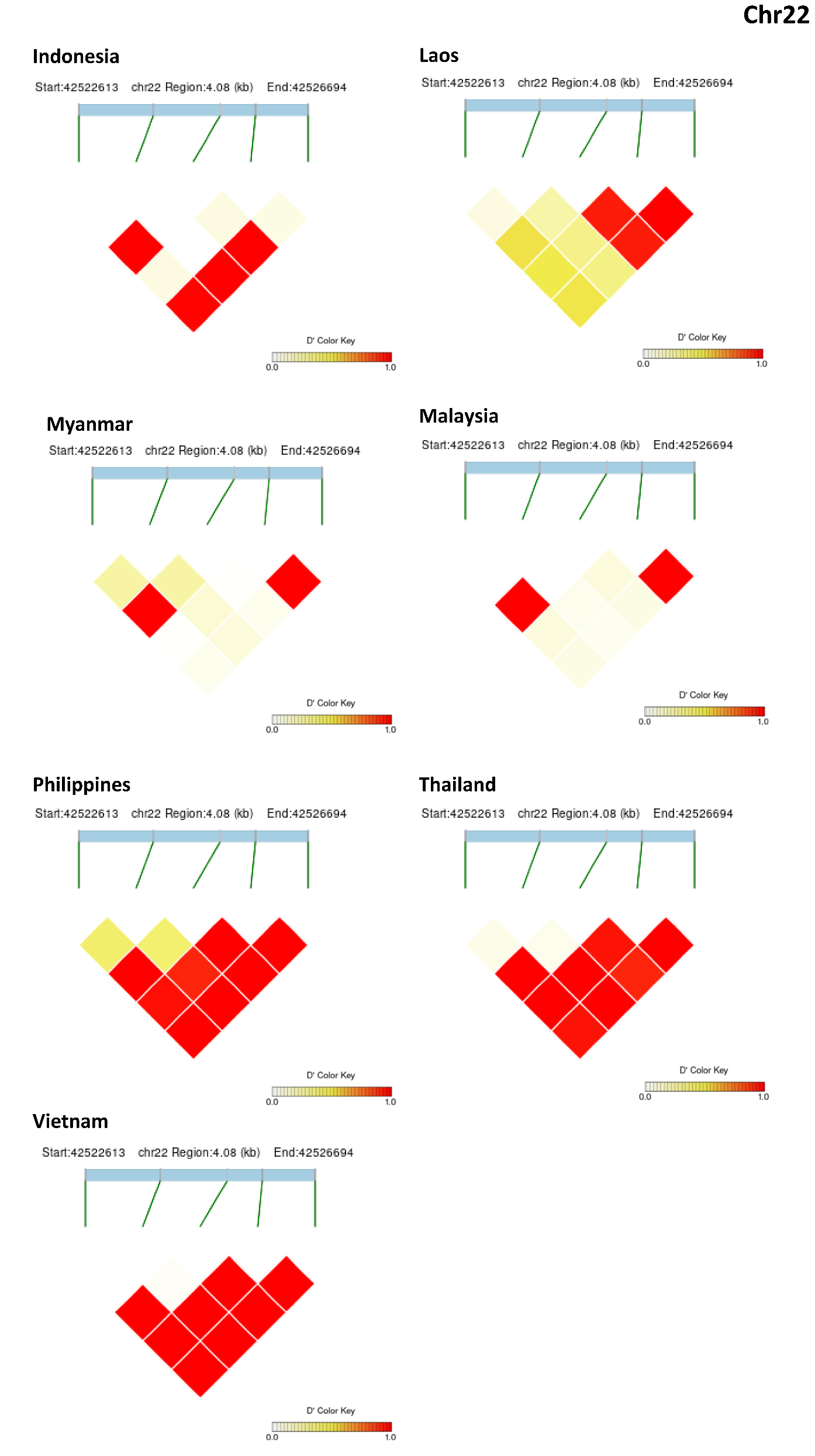


**Supplementary Figure 13**. Linkage disequilibrium plot of the variants within Chromosome 22 in SEA populations. Linkage disequilibrium for SNPs relative within Chromosome 22 (Chr22:42522613-42526694) of seven countries denotes D’ value. There are five SNPs including rs1135840, rs16947, rs1058164, rs1081003 and rs1065852 of *CYP2D6* located in this region.


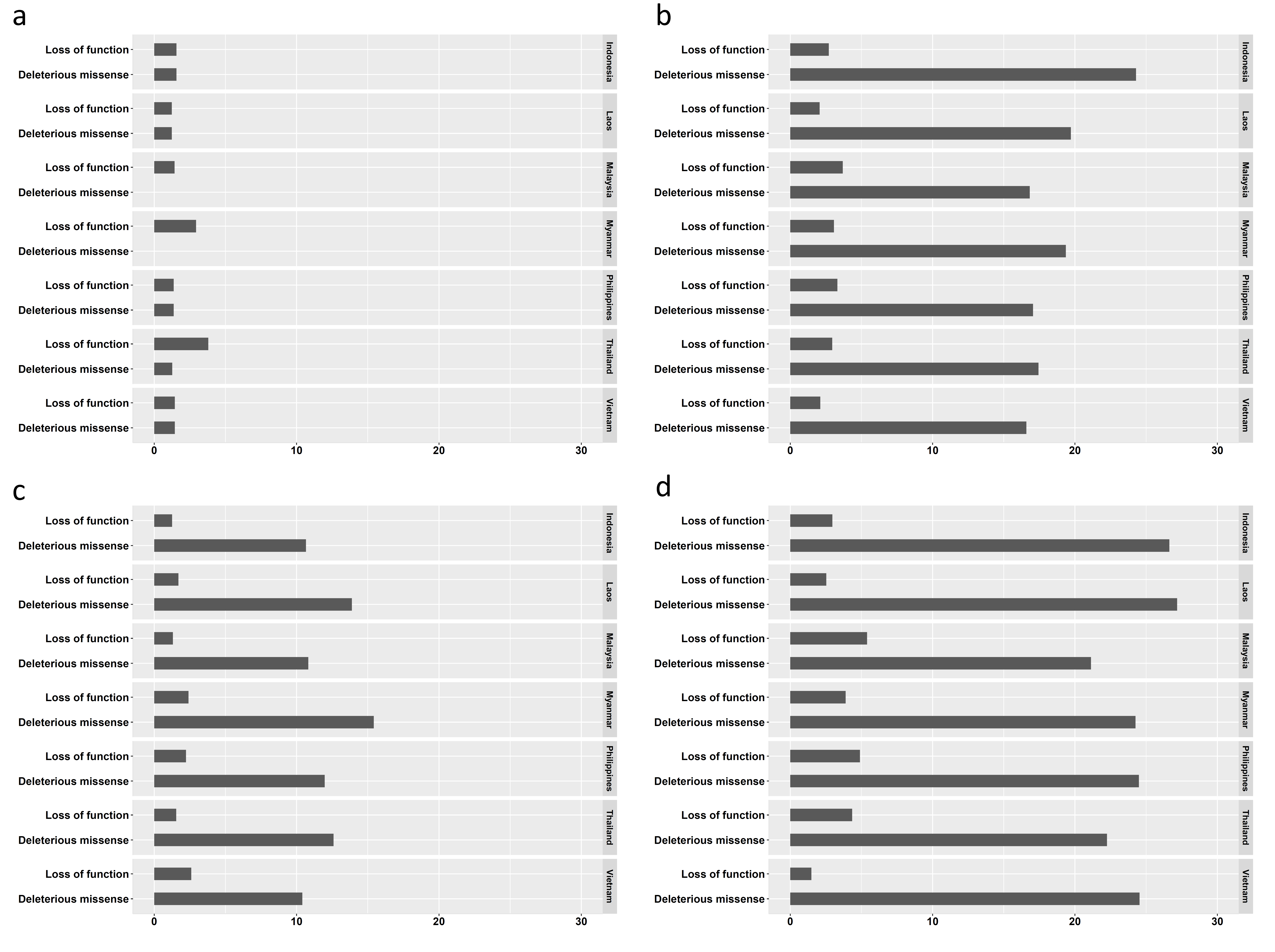


**Supplementary Figure 14**. The proportions of the functional impact of pharmacogenomic variants in SEA populations. a) Major allele frequencies, b) Minor allele frequencies, c) Common allele frequencies, d) Rare allele frequencies.
